# Mechanisms of microbiota-gut-brain axis communication in anxiety disorders

**DOI:** 10.3389/fnins.2024.1501134

**Published:** 2024-12-09

**Authors:** Min Jiang, Li Kang, Ya-Li Wang, Bin Zhou, Hong-Yi Li, Qiang Yan, Zhi-Gang Liu

**Affiliations:** ^1^Department of Clinical Laboratory, Neijiang Central District People’s Hospital, Neijiang, Sichuan, China; ^2^Department of Anesthesiology, The First People’s Hospital of Neijiang, Neijiang, Sichuan, China; ^3^Department of Neurology, Neijiang Central District People’s Hospital, Neijiang, Sichuan, China

**Keywords:** anxiety disorders, gut microbiota, MGBA, neural signal, endocrine pathways, immune pathways

## Abstract

Anxiety disorders, prevalent mental health conditions, receive significant attention globally due to their intricate etiology and the suboptimal effectiveness of existing therapies. Research is increasingly recognizing that the genesis of anxiety involves not only neurochemical brain alterations but also changes in gut microbiota. The microbiota-gut-brain axis (MGBA), serving as a bidirectional communication pathway between the gut microbiota and the central nervous system (CNS), is at the forefront of novel approaches to deciphering the complex pathophysiology of anxiety disorders. This review scrutinizes the role and recent advancements in the MGBA concerning anxiety disorders through a review of the literature, emphasizing mechanisms via neural signals, endocrine pathways, and immune responses. The evidence robustly supports the critical influence of MGBA in both the development and progression of these disorders. Furthermore, this discussion explores potential therapeutic avenues stemming from these insights, alongside the challenges and issues present in this realm. Collectively, our findings aim to enhance understanding of the pathological mechanisms and foster improved preventative and therapeutic strategies for anxiety disorders.

## 1 Introduction

Characterized by persistent unease, fear, and cognitive disturbances, anxiety disorders are highly prevalent, with substantial recurrence rates post-treatment, posing significant public health challenges and affecting socio-economic health (Scholten et al., 2021). For an extended period, the etiology of anxiety disorders has been primarily attributed to neurochemical factors, brain structure, and function, as well as environmental, genetic, and psychological influences. These areas have thus become the main focus of scholarly research. Despite extensive studies, effective treatment outcomes remain elusive. Current treatments frequently result in insufficient symptom relief or significant side effects for many patients. This issue stems from the underappreciated complexity of the mechanisms behind anxiety disorders. Moreover, the development of new therapeutic strategies is hampered by a limited understanding of these mechanisms. Therefore, probing the complex mechanisms underlying these disorders is crucial for devising more potent therapeutic strategies.

Recent interdisciplinary research in neuroscience and microbiology is revealing the complex interactions between the gut and the CNS. The gut microbiota, comprising trillions of microorganisms, is essential for digestion, immune regulation, and metabolism. It also participates in bidirectional communication with the brain via the MGBA, influencing human behavior and psychological states (Cryan et al., 2019). The MGBA is a comprehensive system that includes gut microorganisms, the gastrointestinal tract, the vagus nerve (VN), and endocrine and immune components. It acts as a vital link between the gut microbiome and the brain. Evidence shows that gut-derived stimuli can directly affect brain activity via the VN. Additionally, the metabolic products of gut microbiota can alter endocrine balance and neuroinflammation states, thus affecting the brain’s regulation of cognition and emotion (Generoso et al., 2021).

The role of gut microbiota in regulating emotions and behavior, especially via the CNS, is becoming more apparent as research advances. Numerous studies highlight that the MGBA is the primary communication pathway for this interaction. Factors such as neural signaling, endocrine mechanisms, and immune regulation significantly influence the functioning of this axis. These insights open new avenues for understanding and treating anxiety and other mental disorders, suggesting novel treatment strategies that include manipulating gut microbiota. Techniques such as the use of probiotics, prebiotics, and fecal microbiota transplantation (FMT) can modify the composition of gut microbiota and rebuild the gut environment, improving the psychological well-being of individuals (Baske et al., 2024; Chudzik et al., 2021; Noonan et al., 2020).

This review systematically elucidates the mechanisms through which the MGBA mediates anxiety disorders, focusing on recent advances in neural, endocrine, and immune pathways. Drawing on these findings, we discuss currently available therapeutic strategies, as well as unresolved questions and challenges within this research domain.

## 2 Gut microbiota

The gut microbiota comprises a vast community of microorganisms, including bacteria, archaea, and fungi, inhabiting the human gastrointestinal tract. This microbial population is enormous, vastly outnumbering human cells and boasting a vast genetic diversity that represents about 99% of the genes in our bodies. The gut microbiota evolves alongside the host, mutually influencing each other and performing functions akin to those of the nervous, endocrine, and immune systems (Chen et al., 2021; Cryan et al., 2019). The gut is a stable, resilient ecosystem. Maintaining the balance of this microenvironment is vital for human health, involving microbial population dynamics and changes in the synthesis and metabolism of products that alter intestinal pH, stress levels, and hormone secretion. Disruptions in this stable microenvironment, beyond its self-repair capacity, can lead to diseases, including mental disorders like anxiety. One human study has shown that, compared to healthy individuals, patients with generalized anxiety disorder have a significantly lower abundance and diversity of gut microbiota, indicating a pronounced state of dysbiosis (Jiang et al., 2018). Xiong et al. (2023) have cataloged over 30 basic and epidemiological studies demonstrating the relationship between the gut microbiota and a range of mental disorders, including anxiety. Current research indicates that gut microbiota metabolizes dietary fiber into bioactive components, enhancing digestion and nutrient absorption. Additionally, it produces various antimicrobial substances and other metabolic products through interaction with the host, which may protect beneficial microbiota and the host, and influence mental states and behaviors associated with anxiety disorders (Antushevich, 2020; Gomaa, 2020).

## 3 Anxiety disorders

Anxiety disorders, also known as anxiety neurosis, represent the most prevalent type of neurosis, stemming from the interaction of genetic and environmental factors. They manifest as episodic or persistent anxiety, accompanied by symptoms of autonomic nervous system dysregulation. Beyond emotional disturbances, patients may experience compromised executive functions and cognitive processes. The pathogenesis of anxiety disorders, involving complex dysregulation across multiple systems, remains incompletely understood. Contemporary medical research on the pathogenesis of anxiety focuses on neurotransmitter dysregulation, endocrine imbalances, and immune system dysfunctions. Communication within the CNS depends heavily on chemical neurotransmitters. Research has identified that abnormal concentrations of various neurotransmitters in synaptic clefts can precipitate anxiety symptoms. The neurotransmitters implicated, such as *γ*-aminobutyric acid (GABA), serotonin (5-hydroxytryptamine, 5-HT), dopamine (DA), and norepinephrine (NE), all common in brain regions involved in emotional regulation (Hakamata et al., 2022; Nguyen et al., 2021; Ren, 2016; Xia et al., 2021). Endocrine dysfunctions can lead to psychological and behavioral changes, often triggered by external stimuli affecting the endocrine system, especially the hypothalamic–pituitary–adrenal (HPA) axis (Tafet and Nemeroff, 2020). Additionally, studies indicate that anxiety patients often display immune dysfunctions (Hou et al., 2017). Current pharmacological treatments primarily include first-line medications like selective serotonin reuptake inhibitors (SSRIs) and serotonin-norepinephrine reuptake inhibitors (SNRIs), alongside second-line drugs such as beta-blockers and benzodiazepines. While these drugs are effective in reducing anxiety, they also have drawbacks including delayed onset, variable effectiveness, withdrawal symptoms, and side effects such as headaches, nausea, and sexual dysfunction. Therefore, exploring the complex mechanisms underlying anxiety is critical to developing novel therapeutic strategies (Szuhany and Simon, 2022).

## 4 Gut MGBA-mediated pathways in anxiety disorders

### 4.1 Neural signal pathways

**Figure 1:**
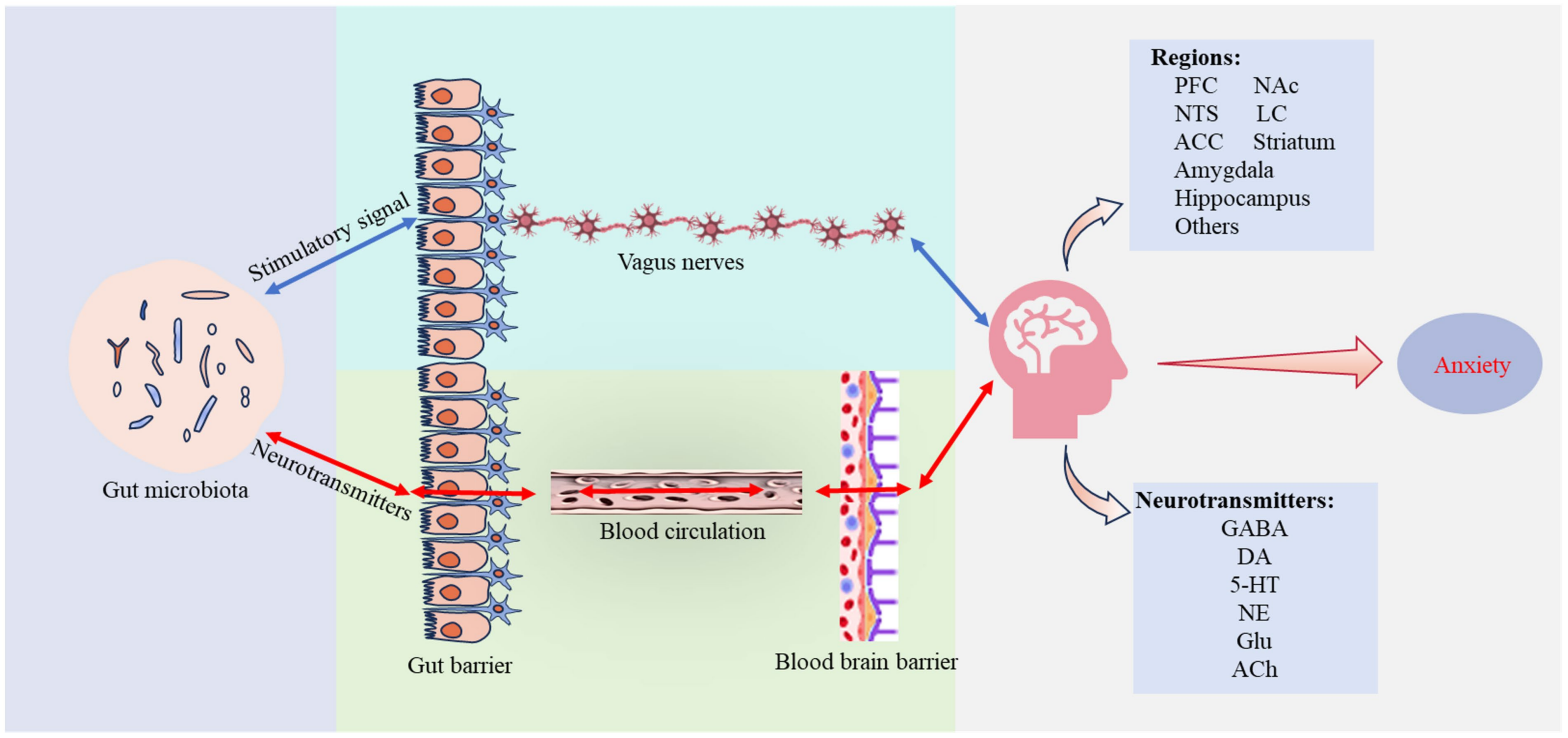
Neural signaling pathways. Gut microbiota contribute to anxiety regulation through two distinct neural pathways: one pathway transmits sensory signals from the intestinal wall to brain regions involved in emotional regulation through the vagus nerve (VN); the other pathway involves neurotransmitters produced by microbial metabolism that traverse the gut and blood–brain barrier (BBB), altering the equilibrium of original neurotransmitters. Both pathways result in altered neurotransmitter levels and changes in the function of brain regions involved in emotional regulation, ultimately affecting the development and progression of anxiety. Supplementary explanation of acronyms used in figure: 5-HT, 5-hydroxytryptamine; ACC, anterior cingulate cortex; Ach, acetylcholine; DA, dopamine; GABA, y-aminobutyric acid; Glu, glutamate; LC, locus coeruleus; Nac, nucleus accumbens; NE, norepinephrine; NTS, nucleus tractus solitarius; PFC, prefrontal cortex.

Neural signaling pathways consist of multiple neurons and synapses that conduct information in various directions to facilitate physiological activities. The VN, containing extensive visceral sensory and motor fibers, is the primary bidirectional regulatory channel connecting visceral organs with the CNS. Gut microbiota metabolize substances including short-chain fatty acids (SCFAs), vitamins, and lipopolysaccharides (LPS), and also synthesize and utilize neurotransmitters such as GABA, DA, NE, and 5-HT (Dicks, 2022; Strandwitz, 2018). These compounds can stimulate the enteric nervous system (ENS) within the gastrointestinal tract wall, converting chemical signals from the gut into neural impulses that are transmitted to the VN and CNS. Additionally, they may cross the blood–brain barrier (BBB) to impact specific brain regions directly. This interaction helps maintain the balance of the gut microbiota and its metabolic products, affecting related neural pathways and influencing behaviors and emotions (Figure 1).

The VN, one of the longest cranial nerves, originates from the medulla oblongata and extends to the thoracic and abdominal regions, terminating beneath the intestinal epithelium. This nerve detects various stimuli from the gut and conveys them to the CNS, influencing behavioral, cognitive, and emotional regulation. The gut microbiota and its metabolic products act as major sources of stimulation, forming a bidirectional connection with the brain via the VN and participating in the modulation of anxiety.

Research by Klarer et al. (2014) demonstrated that subdiaphragmatic vagotomy blocks afferent signals from the gut, resulting in changes in noradrenergic and GABAergic neurons within the ventral prefrontal cortex (vPFC) and nucleus accumbens (NAc), significantly reducing anxiety-like behaviors. Similarly, chronic lesioning of gut-innervating vagal afferent neurons has been found to disrupt GABAergic gene networks in the male rat amygdala, impacting anxiety states (Krieger et al., 2022). These findings highlight the critical role of VN communication in maintaining neurotransmitter system balance essential for anxiety regulation. Other studies show that afferent vagal neuron cell bodies are located in the nodose ganglion (NG) and extend to various visceral organs, including the gastrointestinal tract. They respond to gut stimuli and transmit signals to the nucleus tractus solitarius (NTS) via ascending pathways. Regulation of the locus coeruleus (LC)-NE system in the basolateral amygdala (BLA) via the NTS can mediate anxiety responses, while bilateral ablation of vagal afferents from the gastrointestinal tract can prevent the development of negative emotions (Chen et al., 2023; Cordner et al., 2021). These details underscore the extensive role of the VN and the LC-NE system as a key mediator in transforming gut signals into emotional responses, enhancing our understanding of how visceral information influences CNS activity. However, the mechanisms by which neural networks acquire and integrate these signals are not fully understood. There are indications that harmful metabolic products from the gut microbiota may induce local or systemic chronic inflammation, potentially affecting sensory neurons in the NG, and thus altering vagal nerve function. Although the data is incomplete, these insights are crucial for understanding the importance of gut microbiota-VN communication in the etiology and progression of disorders such as anxiety disorders (Cawthon and de La Serre, 2018).

Furthermore, neurotransmitters including GABA, DA, NE, and 5-HT, metabolized by gut microbiota, may cross the BBB under specific conditions like inflammation, influencing brain regions that regulate anxiety emotions (Strandwitz, 2018). This translocation highlights another pathway through which the gut microbiota can influence the emotional regulation via endogenous neurotransmitters.

*γ*-aminobutyric acid, an inhibitory neurotransmitter within the CNS, plays a crucial role in regulating physiological and psychological responses, especially emotional regulation. *Bifidobacterium* and *Lactobacillus* in the gut microbiota can ferment and produce GABA. This neurotransmitter activates the TrkB receptor for brain-derived neurotrophic factor (BDNF), supporting the integrity and function of the hippocampus. Such activation may alleviate symptoms of stress-induced anxiety and depression while enhancing 5-HT and DA levels (Kim et al., 2024).

5-HT, another inhibitory neurotransmitter, regulates emotions and stress responses by regulating neuronal activity in the amygdala and prefrontal cortex (PFC). Approximately 95% of 5-HT is produced in the gut, with enterochromaffin cells (ECL) as its main source of synthesis (Koopman et al., 2021). These cells produce 5-HT by ingesting cellular debris, microbial entities, and tryptophan (Trp) present in bodily fluids. Moreover, bacterial genera such as Enterococcus, Escherichia, and Lactobacillus can generate 5-HT through the metabolic conversion of Trp, underscoring the gut’s crucial role in regulating body 5-HT levels.

DA is fundamental in regulating brain reward, attention, and motivation, influencing emotional states based on the organism’s perception of reward. Research shows the involvement of various bacterial genera in the gut in regulating DA levels, including *Bacillus*, *Lactobacillus*, and *Enterococcus*. For instance, *Enterococcus faecium* can produce levodopa (L-DOPA), which, upon crossing the BBB, is converted into DA within the brain, potentially at dopaminergic, adrenergic, and serotonergic nerve terminals (Wang et al., 2021). *Bacillus licheniformis* may decrease the levels of Trp, DA, and GABA by modifying the gut’s microenvironment and boosting the production of colonic SCFAs, alleviating symptoms associated with anxiety and depression (Feng et al., 2023). A recent study has also identified a novel mechanism by which gut bacteria regulate DA levels after the methylation of DA by host catechol *O*-methyltransferase (COMT), enabling the conversion of 3-methoxytyramine (3MT) back to DA (Rich et al., 2022). DA also serves as a precursor for the synthesis of NE.

The NE is a key neurotransmitter studied in the context of anxiety disorder pathogenesis. Its levels are affected not only by synthetic precursors like DA but also by certain gut microbiota. For example, heat-killed *Enterococcus faecalis* (EC-12) can boost the concentration of central adrenoceptor b3 (Adrb3) activation in the prefrontal cortex of male mice, enhancing the release of NE. Additionally, Bifidobacterium CECT 7765 has been found to decrease NE levels in the hypothalamus of male mice (Huang and Wu, 2021). These findings support the role of gut microbiota in influencing the synthesis and release of NE. The CNS’s ascending projections of NE originate in the LC of the brainstem and reach multiple brain regions, regulating emotions, arousal, and cognitive functions. For example, activation of the LC-NE system, particularly projections to the anterior cingulate cortex (ACC), is linked to increased anxiety-like behavior in the context of chronic pain (Suárez-Pereira et al., 2022). NE also affects the cardiovascular system by increasing heart rate and vasoconstriction, potentially exacerbating anxiety perceptions.

The cholinergic neuronal system is involved in emotional and affective regulation, and abnormalities may lead to emotional disorders, including depression and anxiety. Acetylcholine (ACh) is secreted by intestinal tuft cells and microbes such as *Lactobacillus*. Most of this secreted ACh is transported via the bloodstream to various tissues and organs, while a small amount enters brain tissue through mechanisms like choline transporters. This suggests that cholinergic signaling may represent another pathway through which gut microbiota influence brain function and emotional regulation, although further empirical evidence is needed to confirm (Hendel et al., 2022; Wiley et al., 2021).

Glutamate (Glu) is an excitatory neurotransmitter critical to the normal functioning of the CNS. The role of Glu in anxiety disorders is still debated, with no clear consensus yet reached. Some researchers believe that abnormalities in Glu metabolism may disrupt the balance of other neurotransmitters, affecting emotional regulation. *Bifidobacterium* and *Lactobacillus* can transform Glu to GABA, influencing the synthesis and release of GABA, and thereby indirectly impacting anxiety levels (Clos-Garcia et al., 2019; Nasir et al., 2020). Additionally, Glu’s interaction with *N*-methyl d-aspartate (NMDA) receptors may regulate neurotransmission and neuronal excitability, contributing to the development and progression of anxiety disorders (Riaza Bermudo-Soriano et al., 2012). Although conclusive data are lacking, existing findings suggest that Glu might be a significant mediator of anxiety affected by gut microbiota.

It is crucial to understand that the array of neural output signals is governed not by a single neurotransmitter but by the interactions of various neurotransmitters within complex neural networks. For example, GABA has an indirect inhibitory effect on the release of DA, 5-HT, and NE, while also reducing Glu excitability. Conversely, 5-HT can modulate GABA release through its receptors. The 5-HT and DA systems maintain a mutual regulatory relationship, affecting DA synthesis, release, and reuptake, especially in areas like the PFC and striatum. DA can either suppress GABA secretion or stimulate Glu release through its receptors. ACh can also inhibit GABA release and enhance DA secretion through interactions with other neurotransmitters, however, the effectiveness of this action depends on the specific brain regions and receptor subtypes involved. In summary, this represents a highly complex sum of effects involving multiple receptors, regions, and signal transduction pathways.

### 4.2 Endocrine pathways

**Figure 2:**
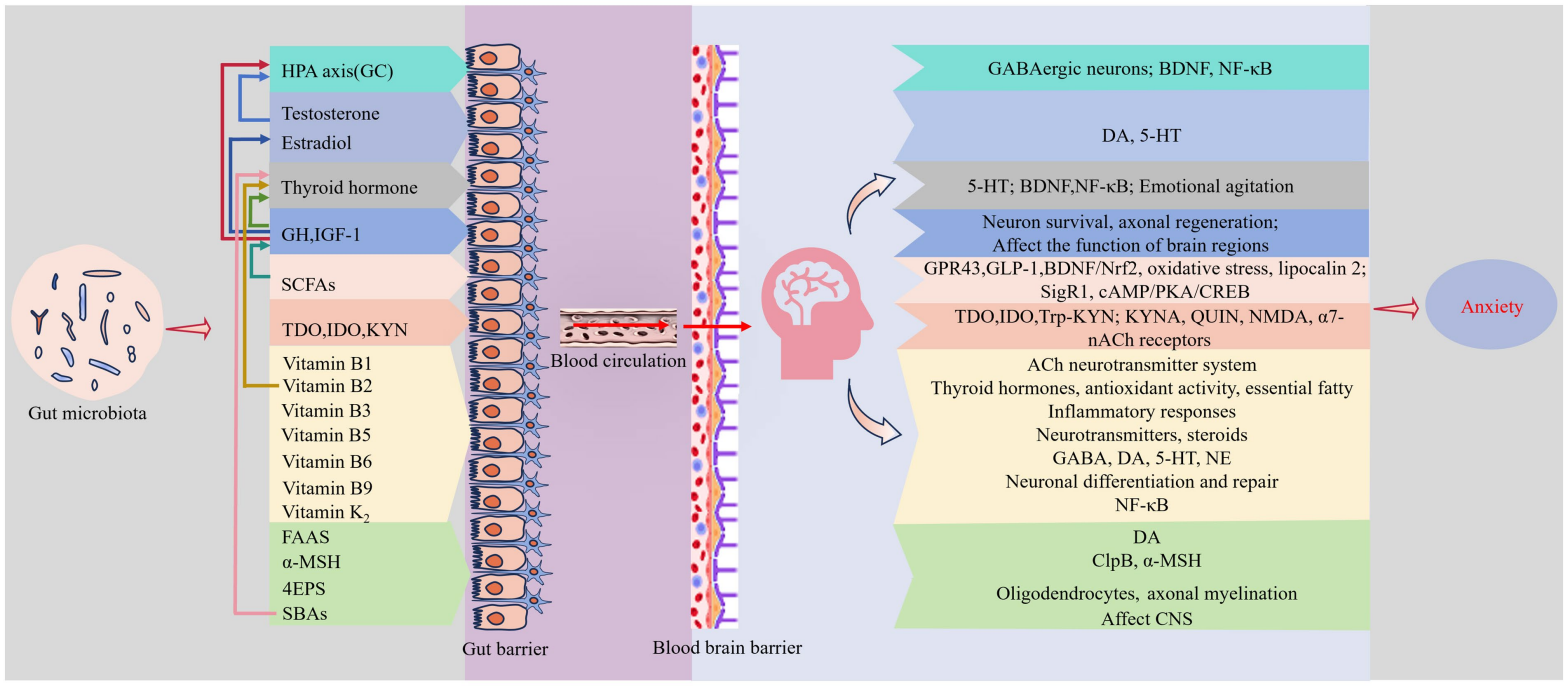
Endocrine pathways. Gut microbiota metabolites, including glucocorticoids (GCs), sex hormones, thyroid hormones, growth hormones, short-chain fatty acids (SCFAs), Trp-2,3-dioxygenase (TDO), indoleamine-2,3-dioxygenase (IDO), kynurenine (KYN), vitamin K2, water-soluble B vitamins, fatty acid amides (FAAs), a-melanocyte-stimulating hormone (a-MSH), 4-ethylphenylsulfate (4EPS), and secondary bile acids (SBAs), can cross the gut barrier and the BBB into specific regions of the brain associated with mood regulation under certain conditions. These metabolites mediate anxiety through diverse mechanisms, and their interactions create a complex and layered system that collectively influences the development and management of anxiety. Supplementary explanation of acronyms used in figure: BDNF, brain-derived neurotrophic factor; ClpB, caseinolytic protease B; CNS, central nervous system; GH, growth hormone; GLP-1, glucagon-like peptide-1; HPA, hypothalamic–pituitary–adrenal; IGF-1, insulin-like growth factor-1; KYNA, kynurenic acid; NMDA, *N*-methyl d-aspartate; QUIN, quinolinic acid; SigR1, Sigmar-1 receptor; a7-nACh, a7-nicotinic ACh.

The endocrine system, a complex network of tissues and glands, synthesizes, stores, and release hormones, neurotransmitters, and cytokines. It plays a key role in regulating growth and development, metabolism, immune response, and emotional changes. Some scholars refer to the human colonizing microbiota as a “virtual endocrine organ,” noting that its synthesis and metabolic products emit chemical signals similar to those of hormones (O'Callaghan et al., 2016). The gut microbiota, the largest reservoir of microorganisms in the human body, participates in regulating anxiety through various endocrine pathways (Figure 2).

The HPA axis is the body’s principal neuroendocrine system and a major bidirectional regulatory pathway of the MGBA. Studies show that gut microbiota can disrupt the normal development of the HPA axis, influencing stress responses, emotions, and cognitive functions. Activation of the HPA axis initiates the synthesis and secretion of glucocorticoids (GCs) from the adrenal glands. These hormones interact with receptors in the hypothalamic paraventricular nucleus (PVN) and the pituitary, reducing the production and release of corticotropin-releasing hormone (CRH) and adrenocorticotropic hormone (ACTH), creating a negative feedback loop. GCs in the hippocampus can suppress HPA axis activity by acting on GABAergic neurons in the PVN (Leistner and Menke, 2020). Research by Frankiensztajn et al. (2020) has shown that mice subjected to restraint stress exhibit increased blood corticosterone levels, decreased BDNF expression in the hippocampus, activated NF-κB, and elevated monocyte recruitment to the colon. Furthermore, the relative abundance of Proteobacteria and *Escherichia coli* increased, while the abundance of *Lactobacillus* significantly decreased, feeding symbiotic *Lactobacillus* significantly improved these anxiety-like behaviors and reduced associated biomarkers (Frankiensztajn et al., 2020). Furthermore, studies on animals indicate that FMT can lower arachidonic acid and raise corticosterone in rats affected by high-salt-induced hypertension (Yan et al., 2020). It is critical to acknowledge that the effects of such transplantation are not immediate. Investigations reveal that germ-free (GF) mice, after receiving gut microbiota from specific pathogen-free (SPF) mice at week six, did not exhibit altered HPA axis responses to stress until week 14, suggesting a critical timeframe for these effects (Neufeld et al., 2011). These findings underscore the complex interplay between the gut microbiota and the HPA axis in regulating stress responses and anxiety-like behaviors.

Extensive research suggests that gut microbiota can impact host sex hormone levels, which may modify anxiety emotions (Liu et al., 2022; Matsushita et al., 2022; Santos-Marcos et al., 2023). *Escherichia coli* in the gut can synthesize 3*β*-hydroxysteroid dehydrogenase (3β-HSD), facilitating testosterone degradation. Administering this bacterium to rats significantly reduced testosterone levels in both blood and brain, triggering negative emotions like anxiety and depression (Li et al., 2022). Moreover, testosterone from peripheral blood is known to cross the BBB, enhancing DA and 5-HT release in the striatum and NAc, thereby attenuating anxiety-like emotions and indicating another pathway through which gut microbes might regulate emotions via testosterone (McHenry et al., 2014). The analysis of clinical cases revealed that women are approximately twice as likely as men to experience anxiety disorders. Although women are more sensitive to testosterone, they produce it at rates approximately one-tenth those of men, potentially explaining the observed gender disparities in anxiety disorder prevalence. Additionally, *Escherichia coli* and other bacteria can metabolize and produce *β*-glucuronidase (GUS), enzymes that influence estrogen levels by promoting the dissociation and hydroxylation of estrogen in the gut (Hu et al., 2023). Fluctuations in estrogen levels are also closely linked to anxiety, as estrogen withdrawal can induce neural plasticity in hypothalamic and dorsal raphe nucleus neurons, increasing anxiety-like behaviors (Hedges et al., 2021). Another study demonstrated that middle-aged female rats displayed anxiety-like states post-ovariectomy, which significantly improved following estradiol supplementation (Renczés et al., 2020). Furthermore, estrogen has been shown to elevate HPA axis activity, further elucidating the gender differences in anxiety disorder prevalence and highlighting the need for personalized treatment strategies based on hormonal status, thereby suggesting that sex hormones may play a role in regulating anxiety through modulation of the HPA axis (Peirce and Alviña, 2019).

A two-sample Mendelian randomization study identified that 34 gut microbiota taxa are involved in regulating thyroid function, thereby affecting thyroid hormone levels (Xie et al., 2023). There is a close link between thyroid hormone levels and the development of anxiety disorders. Studies in animals have demonstrated that levothyroxine-induced hyperthyroidism in rats significantly elevates serum FT3 and FT4 levels, increases brain 5-HT expression, and reduces hippocampal BDNF content, leading to pronounced anxiety-like behaviors. Conversely, hypothyroid rats induced by ^131^I injection exhibited opposite effects (Yu et al., 2015). Clinical cases also demonstrate that an excess of thyroid hormones can cause increased metabolic activity, higher heart rates, and emotional agitation, potentially precipitating or exacerbating anxiety behaviors. Interestingly, some reports indicate that hypothyroidism can also result in symptoms of anxiety. Buras et al. (2014) reported that hypothyroidism may diminish BDNF signaling in hippocampal neuron dendritic spines, resulting in decreased dendritic spine density, which induces anxiety. These conflicting results might relate to the varied impacts of thyroid hormone levels and the state of the thyroid axis on neurotransmitter systems and neural plasticity. While definitive evidence is yet to be established, these findings adequately illustrate the bidirectional nature of thyroid dysfunction and its influence on emotional states, with gut microbiota acting as a potential mediator of this dynamic.

Recent advances in microbial endocrinology have revealed that gut microbiota and their metabolic byproducts play significant roles in the regulation of growth hormone (GH) and insulin-like growth factor-1 (IGF-1) via multiple pathways (Jensen et al., 2020). Research links these hormones closely with anxiety, as demonstrated in both human and animal studies. For example, children and adolescents deficient in GH show a higher prevalence of anxiety, which improves with the administration of exogenous GH (Karachaliou et al., 2021). In mice with anxiety resulting from hypothalamic axon damage, GH treatment enhanced IGF-1 and its receptor expression, supporting the survival and regeneration of hypothalamic neurons after injury, thus reducing anxiety symptoms (Li et al., 2023). Although the underlying mechanisms are intricate and not fully elucidated, two primary explanatory approaches can be identified: First, GH receptors located in key brain regions such as the hypothalamus, amygdala, and bed nucleus of the stria terminalis may influence anxiety directly by altering the function of these areas (dos Santos et al., 2023). Additionally, GH and IGF-1 interact with CRH, its downstream hormones, thyroid hormones and their receptors, and sex hormones, potentially participating in anxiety regulation indirectly through these pathways (Fernández-Pérez et al., 2016; Kucharska et al., 2021; Quaresma et al., 2020).

Short-chain fatty acids, crucial fermentation byproducts of carbohydrate metabolism by gut microbiota, notably include acetate, propionate, and butyrate, which constitute approximately 95% of SCFAs. These compounds are essential for the bidirectional gut-brain communication and act as bacterial signaling molecules influencing cellular activities via G protein-coupled receptors on cell surfaces (Parada Venegas et al., 2019). For instance, SCFAs can promote the release of glucagon-like peptide-1 (GLP-1) by activating GPR43 on intestinal L-cells or serving as endogenous ligands for free fatty acid receptors 2 (FFAR2) and 3 (FFAR3). Given that GLP-1 receptors are prevalent in brain regions like the amygdala and limbic system, linked with anxiety, they represent a promising research area. Agonists of GLP-1 receptors have been shown to reduce anxiety-like behaviors in rodent models, possibly by maintaining BDNF and Nrf2 levels and reducing oxidative stress and lipocalin 2 in the hippocampus (Cawthon and de La Serre, 2018; Sağlam et al., 2022). Simultaneously, SCFAs derived from microbes can also enhance the secretion of mature BDNF by activating the Sigmar-1 receptor (SigR1), thereby ameliorating anxiety and depression-like behaviors (Zhang et al., 2023). Based on the discussion of BDNF’s role in anxiety within this article, it is speculated that BDNF is a pivotal target in the anxiety-regulation pathways mediated by gut microbiota. Additionally, SCFAs may influence GH regulation through the cAMP/PKA/CREB pathway, suggesting another potential mechanism through which SCFAs modulate anxiety (Wang et al., 2013).

Specific gut bacteria, such as *Pseudomonas aeruginosa*, synthesize enzymes including Trp-2,3-dioxygenase (TDO) and indoleamine-2,3-dioxygenase (IDO), which are involved in the Trp-kynurenine (KYN) metabolism pathway. This pathway impacts Trp degradation, thus affecting the synthesis and release of neurotransmitters such as 5-HT, DA, and GABA, which are integral to anxiety regulation (Roager and Licht, 2018). It is noteworthy that comparisons of gender differences reveal heightened IDO activation in females, which may be another key factor contributing to the observed gender disparities in the prevalence of anxiety disorders (Songtachalert et al., 2018). Trp can also be converted to KYN by IDO and TDO enzymes, and various CNS cells metabolize KYN into neuroactive substances. For example, astrocytes convert KYN into neuroprotective kynurenic acid (KYNA), whereas microglia generate neurotoxic quinolinic acid (QUIN). These metabolites significantly influence anxiety-related emotions by activating NMDA and α7-nicotinic ACh (α7-nACh) receptors, affecting multiple nervous systems, including the ENS (Evrensel et al., 2020; Kim and Jeon, 2018; Lim et al., 2017).

Vitamins are crucial nutrients for human health. In addition to external sources, specific gut microorganisms are capable of metabolizing and synthesizing certain vitamins, influencing their absorption and conversion. Metagenomic analysis of human feces has shown that gut bacteria can produce vitamin K_2_ and water-soluble B vitamins (Das et al., 2019). These vitamins traverse the intestinal mucosa and are transported into the brain via specialized mechanisms across the BBB and choroid plexus, playing roles in regulating various biological functions including anxiety. For instance, vitamin B1 is involved in regulating the ACh neurotransmitter system. Vitamin B2 plays a role in the regulation of thyroid hormones, antioxidant activity, and the metabolism of essential fatty acids in brain lipids. Vitamin B3 affects brain inflammatory responses, and vitamin B5 is crucial in synthesizing various neurotransmitters and steroids. Vitamin B6 acts as a limiting cofactor in the synthesis of neurotransmitters such as GABA, DA, 5-HT, and NE. Vitamin B9 deficiency can alter DNA stability, hinder neuronal differentiation and repair, and affect the levels of monoamine neurotransmitters such as DA, NE, and 5-HT by regulating the synthesis of tetrahydrobiopterin (BH4). Certain forms of vitamin K_2_ have been shown to mitigate LPS-induced microglial inflammatory responses by inhibiting the NF-κB signaling pathway (Kennedy, 2016). Although direct connections between vitamins and anxiety have not been conclusively established, the pathways they influence significantly overlap with those involved in the pathogenesis of anxiety disorders, offering new perspectives on the gut microbiota-mediated mechanisms of anxiety and underscoring the importance of gut microbiota in nutritional psychiatry.

Additionally, additional metabolites produced by the gut microbiota are implicated in anxiety regulation. For example, certain Gram-negative bacilli in the gut can produce fatty acid amides (FAAs), which interact with the endogenous cannabinoid receptor CB1, activate sensory nerves in the intestinal wall, and transmit signals to the brain, thereby increasing DA levels in the ventral striatum and influencing anxiety-related emotions (Dohnalová et al., 2022). *α*-melanocyte-stimulating hormone (α-MSH), critical in satiety and anxiety signal transduction, is influenced by caseinolytic protease B (ClpB) produced by *Escherichia coli* and other *Enterobacteriaceae*, highlighting ClpB as a potential mediator in anxiety regulation through gut microbiota (Navarro-Tapia et al., 2021). Animal experiments have found that some gut bacteria in mice can produce 4-ethylphenylsulfate (4EPS), a compound that damages oligodendrocytes, inhibits their maturation, and reduces axonal myelination, leading to anxiety-like behaviors. Treatments that foster oligodendrocyte differentiation may alleviate these negative emotions (Needham et al., 2022). In the enterohepatic circulation, about 5% of bile acids (BAs) are unabsorbed and enter the gut where bacteria transform these primary BAs into secondary bile acids (SBAs) through processes like early deconjugation and 7α-dehydroxylation. These secondary products may enter the CNS via circulation and affect anxiety regulation (MahmoudianDehkordi et al., 2022; Xing et al., 2023). Reports also suggest that SBAs can facilitate the activation of thyroid hormones via the G protein-coupled bile acid receptor 1 (GPBAR1), proposing an additional pathway for their role in anxiety regulation. While the precise mechanisms of these correlations remain to be fully elucidated, these findings affirm the active involvement of gut microbiota in the cerebral regulation of emotional states through their metabolic products (Sasaki et al., 2018). Moreover, new gut microbiota metabolites related to anxiety continue to be discovered and elucidated.

### 4.3 Immune pathways

**Figure 3:**
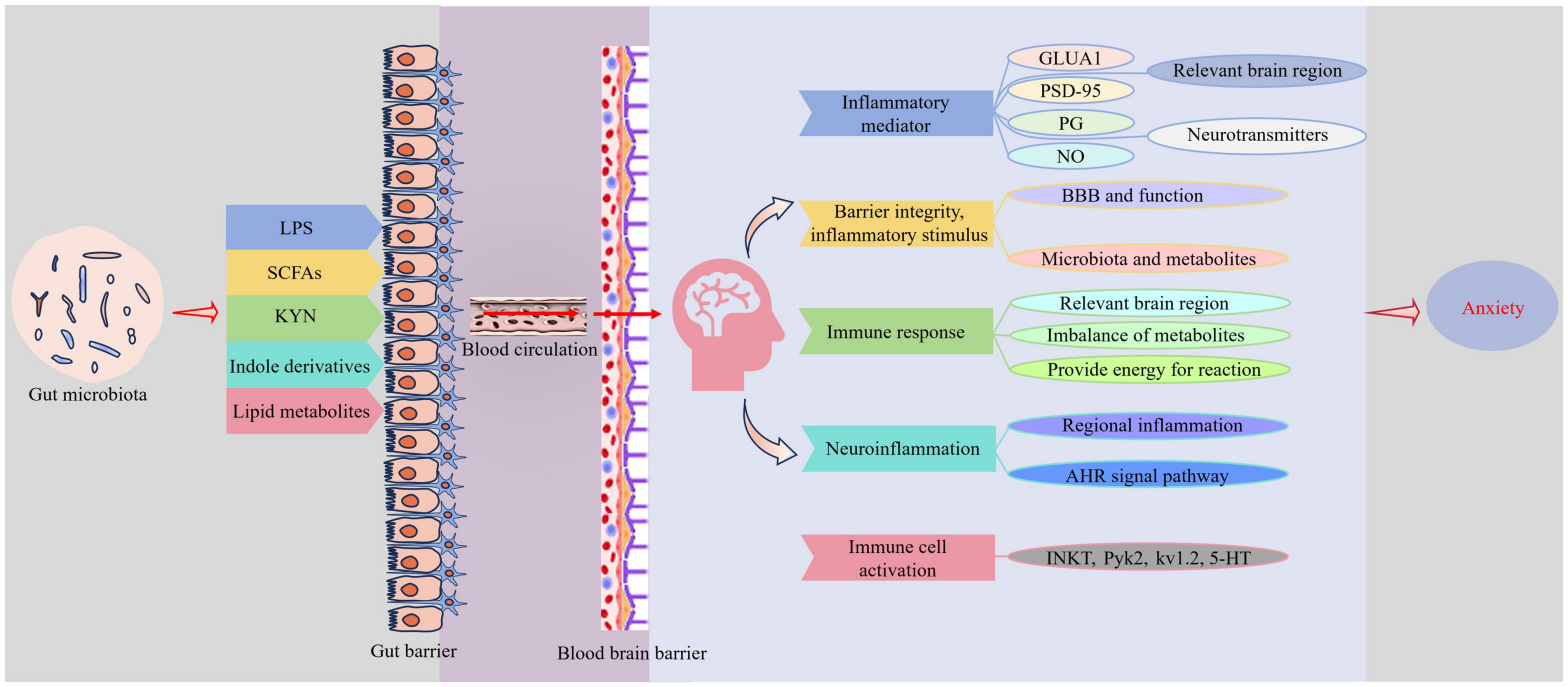
Immune pathways. Pro-inflammatory and anti-inflammatory metabolites produced by gut microbiota are crucial in modulating anxiety emotions through immune pathways. Inflammatory mediators, including lipopolysaccharides (LPS), indole derivatives, and lipid metabolites, can compromise the integrity of the intestinal mucosal barrier, facilitating their entry into the bloodstream and crossing the BBB, thereby initiating neuroinflammation. Conversely, anti-inflammatory agents like SCFAs may counter these effects. The cumulative immune responses of these metabolites affect the function of brain regions and neurotransmitter levels involved in mood regulation, thereby influencing the brain’s ability to modulate anxiety emotions. Supplementary explanation of acronyms used in figure: AHR, aryl hydrocarbon receptor; GluAl, a-amino-3-hydroxy-5-methyl-4-isoxazolepropionic acid receptor A1; INKT, invariant natural killer T; Kv1.2, voltage-gated potassium channel subfamily A member 2; NO, nitric oxide; PG, prostaglandin; PSD-95, postsynaptic density-95; Pyk2, proline-rich tyrosine kinase 2.

The gut, a major organ in contact with the external environment, exhibits distinct regional immune characteristics due to its continuous exposure to diverse antigens and microorganisms. The proper functioning of this system relies on the homeostasis of the gut microenvironment, which comprises intestinal epithelial cells (IECs), mucosa, immune cells, and microbiota. Disturbances in this environment can lead to structural and functional immune disorders. Such disruptions are known to influence brain regions linked with anxiety (Felger, 2018). Historically considered a protective barrier, the BBB was thought to shield the brain from peripheral inflammation. However, recent findings suggest that peripheral inflammation can interact with the brain via signaling mechanisms involving the VN, brain endothelial cells, circumventricular organs (CVOs), and peripheral immune cells. This interaction can trigger neuroinflammation, consequently affecting the brain’s regulatory functions over behavior, cognition, and anxiety (D’Mello and Swain, 2016) (Figure 3).

Lipopolysaccharides, derived from the cell walls of Gram-negative bacteria, are potent pro-inflammatory agents prevalent in the human gut. They are implicated in numerous immune pathways critical to the development and progression of anxiety disorders, primarily through the activation of Toll-like receptors (TLRs), particularly TLR4. TLR4 is expressed extensively across gastrointestinal epithelial cells, immune cells, and neurons. Upon binding of LPS to TLR4, downstream signaling pathways such as MyD88 and NF-κB are activated, contributing directly or indirectly to neuroinflammation in the brain (Carloni and Rescigno, 2023; Peng et al., 2021; Vargas-Caraveo et al., 2017). Research by Yang et al. (2023) has demonstrated that inhibiting the TLR4/MyD88/NF-κB signaling pathway reduces inflammation, protects hippocampal neurons, and significantly alleviates anxiety-like behaviors induced by methyl methanesulfonate in male C57BL/6 mice. This highlights the pivotal role of TLR4-mediated signaling in controlling neuroinflammation and its potential as a target for treating anxiety disorders.

Meanwhile, increased intestinal permeability allows LPS to directly cross the gut mucosa into the bloodstream. This translocation can compromise the BBB, induce neuroinflammation, and affect brain regions responsible for emotional regulation. Accompanying this are releases of pro-inflammatory cytokines, including IL-1β, IL-6, and TNF-*α*. These cytokines are recognized by macrophages and the afferent VN, which activate ascending neural signals to brain areas that manage emotions, precipitating anxiety-like states (D’Mello and Swain, 2016). Additionally, these pro-inflammatory cytokines can downregulate levels of α-amino-3-hydroxy-5-methyl-4-isoxazolepropionic acid receptor A1 (GluA1) and postsynaptic density-95 (PSD-95) in the medial PFC via the subdiaphragmatic VN (Zhang et al., 2020). The regulation of excitability in the PFC through the *N*-cadherin-GluA1 pathway has been observed to mediate anxiety-like behavior in male offspring following prenatal stress (Shao et al., 2021). PSD-95, a critical structural component of the postsynaptic membrane in neurons, is essential for anchoring Glu receptors and maintaining synaptic stability and plasticity. By interacting with relevant receptors, ion channels, and signaling proteins, PSD-95 influences the nervous system’s control over learning, memory, and anxiety (Fitzgerald et al., 2014; Liu et al., 2017).

Receptors for inflammatory factors like TNF-*α*, IL-1β, and IL-6 are also found in brain endothelial cells, neurons, and glial cells. These can bind directly to cytokines in CVOs, which lack a complete BBB, thereby inducing neuroinflammation and subsequent anxiety-like behaviors. Additionally, these pathways can lead to increased production of nitric oxide (NO) and prostaglandins (PGs) in the brain (D’Mello and Swain, 2016). NO can upregulate ACh levels in the NAc, stimulate hippocampal release of NE and Glu, and alter GABA levels depending on its concentration. It also prompts the medial preoptic area to release DA and 5-HT, contributing to anxiety regulation (Gulati et al., 2017). Meanwhile, elevated levels of PGs in regions such as the hypothalamus, hippocampus, and PFC have also been proven to induce anxiety symptoms (Yin et al., 2022). Furthermore, inflammation-induced damage to the BBB can disrupt its regulation and restriction of peripheral neurotransmitters like DA, 5-HT, and NE, thereby affecting anxiety modulation through neural pathways.

The SCFAs serve as energy sources for IECs and microbiota, and in conjunction with thyroid hormones, they reinforce the tight junctions of gut wall cells. This reinforcement potentially affects the gut microenvironment and the integrity of the mucosal barrier. SCFAs also attenuate inflammation by enhancing beneficial gut bacteria and blocking detrimental stimuli like LPS from accessing the systemic circulation (Knezevic et al., 2020; Taylor and Holscher, 2018). Furthermore, SCFAs can traverse the BBB and impact the integrity and function of this barrier. Their receptors are expressed in various cell types, including brain endothelial cells, neutrophils, and lymphocytes. These fatty acids actively participate in anti-inflammatory responses and diminish the production of inflammatory cytokines such as IL-6 and TNF-*α* by inhibiting NF-κB activity, thereby improving anxiety symptoms (Franzosa et al., 2018; O'Riordan et al., 2022). Based on our previous discussions regarding SCFAs, it is evident that SCFAs can regulate anxiety-related emotions through multiple important pathways. This suggests their potential as effective candidate therapeutics aimed at alleviating anxiety symptoms.

In the previous section, we explored how KYN modulates anxiety through the endocrine system. Intriguingly, systemic inflammation triggered by LPS also appears to maintain a complex balance with KYN and its derivatives, which might influence anxiety via the immune system. Many enzymes in the KYN pathway, such as IDO, exhibit immunoreactivity. Pro-inflammatory cytokines including TNF-α, IL-1β, and IFN-*γ* can upregulate IDO expression, facilitating the metabolism of Trp to KYN. TLR4 also activates this pathway, increasing KYN transport to the brain during systemic inflammation. However, some studies suggest that this transport is unrelated to IDO activity. We hypothesize that this effect may result from enhanced permeability of the BBB during inflammation, facilitating the influx of KYN and its metabolites into the brain (Gao et al., 2020; Savitz, 2019). Additionally, certain metabolites in the KYN pathway, such as KYNA and QUIN, have contrasting effects on neurotransmitters. Inflammation might alter their levels, affecting anxiety symptoms. Notably, QUIN can also convert to nicotinamide adenine dinucleotide (NAD+), involved in cellular energy metabolism, potentially influencing immune responses that demand substantial energy, thus connecting gut microbiota with anxiety regulation through an immunological pathway (Platten et al., 2019).

Trp metabolism by gut microbiota produces indole derivatives such as indole-3-acetic acid (IAA) and indole-3-propionic acid (IPA), which may regulate anxiety via immune mechanisms. Studies by Jennis et al. (2017) showed that IPA reduces intestinal permeability exacerbated by a high-fat diet in mice. *In vitro* tests indicate that IPA decreases cytokine-induced permeability in T84 cell monolayers (Jennis et al., 2017). These effects suggest that IPA enhances gut barrier function, limiting harmful substances like LPS from entering the bloodstream and reducing systemic inflammation, thereby improving anxiety symptoms. Furthermore, the aryl hydrocarbon receptor (AHR), involved in Trp metabolism, is expressed in microglia, astrocytes, and neurons in the brain. Indole derivatives like IAA and IPA, as AHR ligands, can cross the BBB and activate AHR signaling in astrocytes, inhibiting neuroinflammation and potentially regulating anxiety (Gao et al., 2020).

Lipid metabolites produced by gut microbiota may regulate anxiety via immune pathways. Recent research suggests that these metabolites are recognized by CD1d molecules on IECs, activating invariant natural killer T (iNKT) cells, which in turn trigger proline-rich tyrosine kinase 2 (Pyk2). Pyk2 activation influences voltage-gated potassium channel subfamily A member 2 (Kv1.2) through tyrosine phosphorylation, reducing K^+^ conductance and increasing Ca^2+^ influx, thereby promoting 5-HT release. Meanwhile, inflammatory states in the gut increase gut barrier permeability, enhancing the translocation of 5-HT into the bloodstream and its entry into specific brain regions involved in anxiety regulation Although further studies are needed to confirm these observations, they introduce a novel perspective for understanding how gut microbiota might control anxiety-related emotions through immune pathways (Luo et al., 2023).

## 5 Current treatment and prevention strategies

The MGBA is an essential pathway through which gut microbiota influence anxiety-related emotions. The initial step in this axis involves the gut microbiota, which normalize the axis by sustaining a stable intestinal microenvironment through appropriate quantities, proportions, and metabolites. Various therapeutic approaches have been employed for the reconstruction of the gut microenvironment, potentially serving as effective strategies to ameliorate anxiety.

Probiotics, which are beneficial bacteria for the gut microbiota, can be administered through oral supplements or included in dietary choices. Popular probiotics include strains of lactic acid bacteria, bifidobacteria, and yeast. These organisms colonize the gastrointestinal tract, engage in interactions with other microbes, augment populations of beneficial bacteria, and suppress deleterious bacteria, thereby promoting a balanced gut microbiota and enhancing the overall gut environment. Prebiotics are food components utilized by gut microorganisms to promote the growth and activity of beneficial bacteria. Typical prebiotics include fructans and glucose-derived oligosaccharides. Although not absorbed by the human body, prebiotics are fermented by beneficial gut bacteria, producing advantageous metabolites like SCFAs, which are crucial for the growth and vitality of beneficial microbes, thus maintaining microbial equilibrium in the gut (Kim et al., 2019; Rau et al., 2024). Probiotics and prebiotics exhibit a synergistic effect and often show greater therapeutic effects when used together. It should be noted that different probiotics and prebiotics may have variable effects among individuals, therefore, individualized selection and use are necessary.

A broad analysis of studies connecting nutrition to mental health disorders reveals that dietary interventions can exert therapeutic effects on chronic neurological conditions. The gut microenvironment serves as a crucial link between the two. Dietary influences on gut microbiota ecological balance primarily involve the composition of microbial communities, gut permeability, inflammatory responses, and the metabolites produced by different microbial populations (Navarro-Tapia et al., 2021; Ross et al., 2024; Schnorr and Bachner, 2016). For instance, diets rich in omega-3 fatty acids, such as those including fish oil, can enhance beneficial microbial communities, bolster gut barrier function, and decrease levels of circulating LPS, thus mitigating systemic inflammation. Additionally, the levels of trace and macronutrients in food can impact the gut microenvironment and microbial composition. A magnesium-deficient diet may reduce beneficial gut bacteria and increase negative emotions, while a high-fat diet can alter gut microbiota composition, affecting metabolism, gut permeability, and inflammation. Moreover, food additives significantly impact the composition of gut microbiota; for instance, high-salt diets may reduce populations of beneficial bacteria such as bifidobacteria, and emulsifiers may increase the pro-inflammatory potential of certain gut microbes (Bear et al., 2020). It is crucial to recognize that these relationships are typically non-linear, thus necessitating careful exploration of optimal dosages when employing beneficial supplements for treatment purposes.

The FMT is a technique employed to reconstruct the gut microenvironment by transferring microbiota from the feces of healthy donors into the patient’s gastrointestinal system. This method helps restore microbial diversity and balance, promotes the growth of beneficial bacteria, and inhibits harmful bacteria. FMT has proven effective for treating recurrent *Clostridium difficile* infection (D and Venkatesh, 2023; Minkoff et al., 2023). However, further research are required to verify its safety and effectiveness in treating other conditions, including anxiety disorders, and to resolve associated technical and procedural challenges.

The reconstruction of the gut microenvironment is a prolonged process, and maintaining reasonable lifestyle habits is necessary for both prevention and during reconstruction. For example, the adverse effects of stress on the gut microbiota can be alleviated by ensuring sufficient rest, engaging in relaxation training, and receiving psychological counseling. Moderate physical activity enhances intestinal motility and improves the gut microenvironment. Furthermore, avoiding the prolonged, frequent, and inappropriate use of antibiotics can minimize their negative impacts on gut microorganisms. Additionally, reducing the use of germicides and excessive disinfection, maintaining moderate hygiene standards, and avoiding the destruction of beneficial microbes are also vital for sustaining a healthy gut microbiota (Fishbein et al., 2023; Gubert et al., 2020; Sciurba et al., 2021).

## 6 Unresolved issues, challenges, and future research priorities

While considerable advancements have been made in the study of the MGBA, numerous challenges persist in this area. The diversity and individual variability of gut microbiota complicate the identification of specific microbial profiles linked to anxiety. Concurrently, most evidence comes from animal studies, and translating these findings to humans requires careful consideration and validation. The precise mechanisms underlying the interactions between microbiota, the gut, and the brain are still not entirely comprehended, and clarifying the directionality of these interactions remains a significant task. Despite these challenges, the active nature of this research field continues to yield new insights, providing hope for further clarification of the complex relationships between gut microbiota and anxiety.

Future research should focus on human clinical trials to validate phenomena observed in animal models, enhance the clinical relevance of findings, and establish causal relationships between gut microbiota and anxiety. Further research is also needed to understand how dietary choices, antibiotics, prebiotics, and probiotics influence gut microbiota and impact the gut-brain axis. Investigating individual differences in gut microbiota composition, influenced by genetic factors and early-life experiences, may offer personalized strategies for managing anxiety through gut microbiota modulation. Additionally, the development of new therapies targeting the MGBA, including psychobiotics, will require rigorous clinical trials to assess their efficacy and safety in treating anxiety disorders. Addressing these challenges will be crucial for leveraging the potential of the MGBA in the prevention and treatment of anxiety and other neuropsychiatric disorders.

## 7 Conclusion

Research increasingly confirms that the MGBA constitutes a bidirectional communication network crucial for maintaining gut physiological homeostasis and influencing brain function and behavior. Neurologically, gut microbiota and their metabolites directly interact with the brain through the VN or indirectly affect emotion-regulating brain regions by crossing the BBB, mediated by neurotransmitters and their precursors derived from bacterial metabolism. In the endocrine pathway, gut microbiota play a pivotal role in the regulation of various hormones and other substances that contribute to stress response and emotional regulation. Immunologically, disruptions in the gut microenvironment can enhance intestinal permeability, permitting the entry of bacterial components and detrimental metabolites into the bloodstream. This influx triggers systemic inflammation, which in turn impacts the nervous system’s role in managing anxiety-related emotions. These insights provide a refined and comprehensive understanding of the pathological mechanisms that underlie anxiety disorders, thereby underscoring potential therapeutic targets and furnishing a more detailed framework for grasping the origins of anxiety and identifying prospective treatment options.

It is important to note that the neuro, endocrine, and immune pathways within the MGBA interact in a complex and intertwined system. The CNS reacts to gut-derived stimuli by activating the endocrine system, which in turn alters hormone levels and other substances, affecting emotional states. Conversely, changes in the endocrine environment can modulate neural activity, influencing neurotransmitter dynamics and emotional regulation. Additionally, systemic inflammation within the immune system can modify neural signaling and endocrine functions, ultimately creating a feedback loop that impacts anxiety responses. This complex interplay suggests that modifications in one pathway can trigger cascading effects on others, underscoring the need for therapeutic strategies that consider the overall effects.

In summary, this study focuses on the unique and interconnected complex mechanisms of the MGBA in the pathogenesis and progression of anxiety disorders, offering new insights into the pathophysiological mechanisms of anxiety and the development of novel therapeutic strategies.
